# *MEIS2* (15q14) gene deletions in siblings with mild developmental phenotypes and bifid uvula: documentation of mosaicism in an unaffected parent

**DOI:** 10.1186/s13039-021-00570-1

**Published:** 2021-12-20

**Authors:** Bin Zhang, Michel Liu, Chin-To Fong, M. Anwar Iqbal

**Affiliations:** 1 Departments of Pathology and Laboratory Medicine, University of Rochester Medical Center, 601 Elmwood Ave, Box 608, Rochester, NY 14642 USA; 2 Department of Pediatrics, University of Rochester Medical Center, 601 Elmwood Ave, Rochester, NY 14642 USA; 3 Renal-Electrolyte and Hypertension Division, Perelman School of Medicine, University of Pennsylvania, Philadelphia, PA 19104 USA

**Keywords:** *MEIS2*, Deletion, Mosaicism, Dosage effect, Orofacial clefts, Developmental delay, FISH, Chromosome microarray

## Abstract

*MEIS2* (Meis homeobox 2) encodes a homeobox protein in the three amino acid loop extension (TALE) family of highly conserved homeodomain-containing transcription regulators important for development. *MEIS2* deletions/mutations have been associated with cleft lip/palate, dysmorphic facial features, cardiac defects, as well as intellectual disability at a variable severity. Here we report on one familial case that two affected siblings carry the same non-mosaic ~ 423 kb genomic deletion at 15q14 encompassing the entirety of *CDIN1* and the last three exons (ex. 10, 11, 12) of the *MEIS2* gene, while their unaffected father is mosaic for the same deletion in about 10% lymphocytes. Both siblings presented with mild developmental delay and bifid uvula, while no congenital cardiac abnormalities were identified. The elder sister also showed syncopal episodes and mild speech delay and the father had atrial septal defects. This is the first report showing multiple family members inherit a genomic deletion resulting in a *MEIS2* partial truncation from a mosaic parent. Taken all together, this study has important implications for genetic counseling regarding recurrence risk and also points to the importance of offering *MEIS2* gene tests covering both point mutations and microdeletions to individuals with milder bifid uvula and developmental delay.

## Introduction

Orofacial clefts (OFCs) are among the most common human birth defects, affecting ~ 1.56 per 1000 live births in North America [1], and present as a broad spectrum of phenotypes ranging from subclinical forms such as bifid uvula, to submucous cleft palate and velopharyngeal insufficiency, and to overt cleft palate. Sporadic OFCs are considered as a multifactorial polygenic trait caused by a number of genetic and environmental factors. Syndromic OFCs are associated with developmental delay, dysmorphic features, or other major congenital anomalies, and mostly have a single genetic cause, either chromosomal or monogenic [2].

*MEIS2* (Meis homeobox 2) is among the recently-identified OFC genes, and its disruption is considered to be the main causative factor contributing to the pathogenesis of chromosome 15q14 deletion syndrome [2–6]. *MEIS2* encodes a homeobox protein in the three amino acid loop extension (TALE) family of highly conserved homeodomain-containing transcription regulators. It is expressed during early fetal brain, forelimb buds, developing hearts, and developing palatal shelves [7–9], and have been shown to be important for cranial and cardiac neural crest development [10, 11]. MEIS2 protein may not be directly involved in complex DNA-binding, and most likely acts as PBX and HOX protein cofactors to form dimeric or trimeric complexes to enhance DNA binding specificity and affinity, and therefore regulate target gene expression [9]. It has been established that haploinsufficiency of the *MEIS2* gene cause a syndromic form of OFCs, often presenting with cardiac defects, facial dysmorphism, as well as intellectual disability at a variable severity [2, 3, 6, 12–14]. *MEIS2* mutations can vary as genomic microdeletions, single nucleotide variants (SNVs), or small insertions and deletions (indels), and most mutations occur de novo [2, 15]. Rare disease-causing mutation mosaicism has been reported [3]. Some *MEIS2* missense mutations have been shown to cause more severe phenotypes than deletions, suggesting a possible dominant negative effect [12, 14]. Multiple transcripts and alternative transcription start sites have been identified and studied for their potential regulatory function during development [16]. However, genotype and phenotype correlation studies between 3′ deletion and the entire gene deletion have not been conducted.

Here, we present one family with a 423 kb deletion encompassing the last three exons of the *MEIS2* (15q14) gene in two siblings with bifid uvula and mild developmental delay and the same deletion as a mosaic lesion in the father with a congenital atrial septal defect. These findings could have implications for disease recurrence risk assessment and genetic counseling. We reviewed the literature and analyzed different mutation types, including loss-of-function microduplications/microdeletions and point mutations, along with the phenotypes associated. This study also points to the importance of offering *MEIS2* gene tests covering both SNVs/indels and microdeletions to individuals with milder bifid uvula and developmental delay.

## Materials and methods

### Chromosome microarray (CMA)

Chromosome array comparative genomic hybridization (aCGH) experiments were performed using the SurePrint G3 Human CGH + SNP Microarray 4 × 180 K (Agilent Technologies, CA), which contains approximately 110,712 oligonucleotides (60mers) for the detection of copy number variations (CNVs), along with 59,647 SNP probes for genotyping and detection of long stretches of contiguous homozygosity. A human genomic male/female reference DNA sample, supplied by Agilent Technologies, is used as same sex controls for each analysis. DNA was extracted from the patient’s peripheral blood using QIAamp® DNA Blood Mini Kit (Cat # 51104, Qiagen Inc., Valencia, CA). Arrays were prepared in accordance with the manufacturer's instructions with an input amount of 500 ng of genomic DNA. Data were analyzed and visualized using the CytoGenomics v5.1.2 software (Agilent Technologies). The threshold for log2 ratios were − 0.25 for losses and + 0.25 for gains. The genomic linear positions are given relative to the GRCh37/hg19 genome assembly.

### Fluorescence in situ hybridization (FISH)

Peripheral blood samples were cultured using standard cytogenetic methods for 72 h with phytohemagglutinin (PHA) stimulation. Fluorescence in situ hybridization was performed with standard techniques using the RP11-450G24 bacterial artificial chromosome (BAC) probe (SpectrumGreen, Empire Genomics, NY) at 15q14 and the TelVysion probe for the subtelomeric region of chromosome 15: WI-5214(D15S936) (SpectrumOrange, Abbott Laboratories, Des Plaines, IL).

### Clinical report

**Fig. 1:**
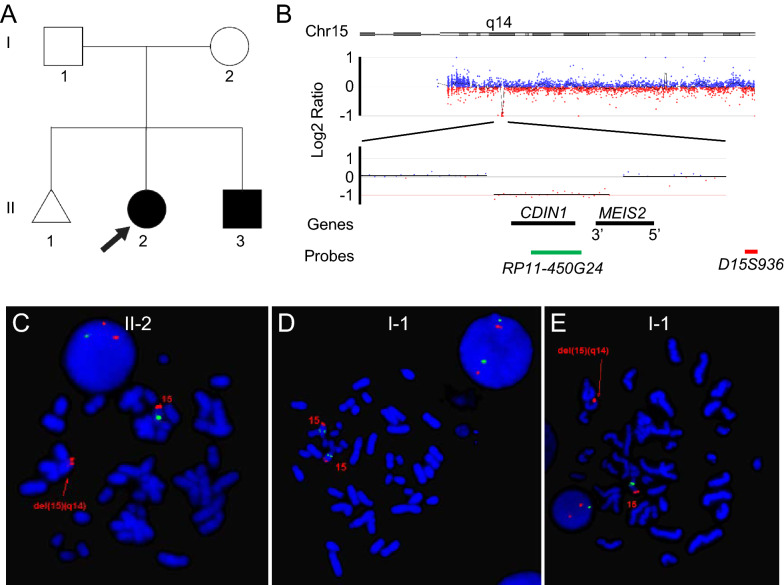
A familial case of 3′ *MEIS2* deletion. **a** Pedigree of the family with deletion of 3′ *MEIS2*; black box indicates subject with speech delay and bifid uvula; arrow indicates the proband. **b** CMA analysis revealed an approximately 423 Kb interstitial deletion of the long arm of chromosome 15 in the proband (II-2). The *CDIN1* gene is deleted, and 3′ part of *MEIS2*, including exons 10, 11, 12 of the NM_170676.5 transcript, is deleted. Colors and locations of FISH probes are indicated. **c** FISH analysis confirmed the deletion in the proband (II-2) and her brother (data not shown). **d**, **e** FISH analysis revealed mosaicism of the same deletion in the father [I-1; **d** normal metaphase and interphase cells; **e** abnormal metaphase and interphase cells with del(15)(q14q14)]

The proband (II-2 in Fig. 1a) was then a 3-year-old female and was referred to the University of Rochester Medical Center (URMC) genetics clinic for evaluation of developmental delay and bifid uvula. Her prenatal history is unremarkable except for intrauterine growth restriction (IUGR). She was born with a full-term gestation. Her birth weight is 2.69 kg (8.85 percentile, Z-score − 1.35) and her birth length is 48.26 cm (33.51 percentile, Z-score − 0.43). She never crawled, scooted around at one year of age, or walked at 18 months. She had mild speech delay and talked clearly with words around 14–15 months. Now she speaks in full sentences. Her growth is adequate despite initial IUGR and she has global developmental delay.

She has a history of recurrent ear infections and had tympanostomy tubes placed. She started to have intermittent syncopal episodes lasting 3–5 s at 1.5 years of age. Her electrocardiogram (ECG) is normal with a normal QTc. An echocardiogram confirms normal cardiac anatomy and function with an intact atrial septum. She has no cardiac abnormalities and her previous prominent foramen ovale flap noted in utero was interpreted as a normal variant. The syncopal episodes of a short duration are not considered to represent a primary cardiac event.

The brother (II-3 in Fig. 1a) was 12 months old at the time of referral and presents with mild developmental delay and bifid uvula. His prenatal history is unremarkable except for oligohydramnios. He was born with a gestational age of 36w5d. His birth weight is 2.58 kg. (5.69 percentile, Z-score − 1.58) and his birth length is 48.26 cm (22.88 percentile, Z-score − 0.74). He did not start walking until he was about 21 months old. He is the 5th percentile for weight. He has slight genu valgum bilaterally. There is no other congenital malformation. He is small but growing steadily along his own growth curve. There is no history of cyanosis, shortness of breath, exercise intolerance, fatigue, tachycardia, diaphoresis, syncope, or presyncope. There is no history of seizures.

The father (I-1 in Fig. 1a) had an atrial septal defect per report. There are no family history of developmental delay and bifid uvula.

## Results

Chromosome microarray analysis revealed a non-mosaic ~ 423 kb genomic deletion at 15q14 (chr15:36808211-37231497) encompassing the entirety of *CDIN1* and 3′ end of the *MEIS2* gene (NM_170676.5) in the proband (II-2) and her younger brother (Fig. 1a, b). The breakpoint defined between chr15:37231467 (the first deleted 3′ probe) and chr15:37285154 (the first undeleted 3′ probe) is within intron 9 of the *MEIS2* gene and causes deletion of last three exons (ex. 10, 11, 12) of the NM_170676.5 transcript. FISH analysis with a BAC probe (RP11-450G24, chr15:36960019-37122831) confirmed the deletion (Fig. 1c). The father is mosaic for the same deletion in 12% (3/25) metaphase and 9% (18/200) interphase cells of his peripheral blood (Fig. 1d, e).

## Discussion

*MEIS2* mutations, including genomic microdeletions and SNVs and indels, have been recently identified to cause syndromic phenotypes, including palatal defects, congenital cardiac defects, and intellectual disability at variable severity and penetrance [2, 6, 14]. We report a genomic microdeletion affecting the last three exons (ex. 10, 11, and 12) of the *MEIS2* gene (NM_170676.5) and *CDIN1* in two siblings, presenting mild developmental delay and bifid uvula. The father is mosaic for the same deletion, but did not present with the syndromic phenotypes. The *CDIN1* gene encodes a novel restriction endonuclease, belonging to the Holliday junction resolvase family, and two missense mutations have been identified to be responsible for autosomal recessive congenital dyserythropoietic anemia type Ib (OMIM #615631) [17]. There are no evidence that heterozygous missense mutation or deletion of the *CDIN1* gene causes disease in the literature. The pLI score, the probability of intolerance to a Loss of Function (LoF) mutation, is 0 for the *CDIN1* gene per the gnomAD database, indicating that heterozygous deletion is likely tolerated and not involved in the phenotype of the siblings of this study. Haploinsufficiency of *MEIS2* with the pLI score of 1 is considered to be responsible for a syndromic phenotype with cleft palate, intellectual disability, heart defects, and dysmorphic features [2]. Up to date, 20 unrelated patients with *MEIS2* mutations and 29 unrelated patients with deletions or exonic duplications have been identified by sequencing analysis and array-CGH analysis [2, 14, 15, 18–21]. Interestingly, a recent genotype and phenotype correlation study showed that *MEIS2* missense variants are associated with a phenotype that overlaps but is broader than that reported in individuals with gross deletions of *MEIS2* [14], suggesting a dominant negative effect. In contrast, a few individuals with genomic microdeletions present a very mild phenotype [22].

*MEIS2* is an evolutionary-conserved homeobox gene, encoding a homeodomain (HD)-containing transcriptional activator regulating cell proliferation and differentiation of various tissues and organs during development [23, 24]. Multiple transcript isoforms of *MEIS2*, of particular, the 3′ splice variants involving exons 11 and 12, have been identified to form four major protein isoforms with different C termini in human [16], which can assemble largely non-overlapping interactomes, allowing them to recruit different proteins to the regulatory regions of target genes [25]. Interestingly, a truncated HD-less isoform of *hth*, the *MEIS2* homolog in drosophila, is expressed alongside the canonical, full-length isoform, can function without directly binding DNA [26], implicating that human *MEIS2* may function similarly when the HD domain is completely or partially truncated. 3′ *MEIS2* truncating microdeletions could cause different phenotypic severity depending on the number of 3′ *MEIS2* exons deleted.

The last three exons (ex. 10–12) of *MEIS2* were deleted in two siblings reported in this study (Table 1). Two other cases in the literature were identified to have deletions encompassing the last three or fewer exons (Patient A and Patient H in [2], Table 1). Interestingly, all four cases did not show congenital cardiac defects, which occur in 72% (13/18) of individuals with *MEIS2* mutations (SNVs or indels), and 38% (10/26) of other microdeletion and duplication cases. The two siblings of this study only present mild developmental delay and bifid uvula, while patient A and patient H didn’t show palate defects, which occur in 80% (16/20) of *MEIS2* mutations, and 85% (22/26) of other microdeletion and duplication cases (Table 1). The average walking age for these four cases are 24mo, which is not significantly different from 26mo for cases with *MEIS2* deletion and a range of 14mo-4y for cases with microdeletion and duplications (Table 1). Only one of four cases presented with dysmorphic facial features, compared to 100% (17/17) of *MEIS2* mutations, and 83% (10/12) of other microdeletion and duplication cases (Table 1). In addition, patient A and patient H have intellectual disability (ID), while two siblings of this study were not evaluated for ID due to their age. It needs to be pointed out that patient H has a 2.92 Mb deletion (chr15:34308789-37231638) encompassing a number of protein coding genes which likely contribute to phenotypes of facial dysmorphism and ID (Table 1).

**Table 1:** Clinical features associated with different *MEIS2* variants

	*MEIS2* dels/dups^#^	*MEIS2* mutations^&^	Patient A^#^	Patient H^#^	II-2; II-3	I-1
Mutation size/coordinates^$^	Variable	SNVs and indels	194 Kb/chr15:36989551_37184183	2.92 Mb/chr15:34308789_37231638	423 Kb/chr15: chr15:36808211-37231497	
					Non-mosaic	Mosaic
Mutation types	Disruption including exon 9*	7 nonsense; 6 missense; 3 splicing; 2 in-frame del; 2 frameshift mutation	Exon 12 deletion*	Exon 10–12 deletion*	Exon 10–12 deletion*	
Inheritance Mode	19 de novo; 2 familial;8 NA	18 de novo/2 pat	de novo	de novo	pat	de novo
Palate defects	22/26; 1 NA	16/20	−	−	Bifid uvula	−
Cardiac defects	10/26; 1 NA	13/18; 2 NA	−	−	−	ASD
Developmental delay	+	+	+	+	Mild	−
Walked@	14mo-4y	18mo (Giliberti et al. 2020); 26mo (11 cases reviewed in Verheije et al., 2019); 30mo (Santoro et al. 2021); 32mo (Su et al. 2020); NA (6 cases from Srivastava et al. 2018, Hildebrand et al. 2020, Douglas et al. 2018)	27mo	30mo	II-2: 18mo; II-3: 21mo	−
Facial features	Variable: 10/12 (14 cases from Verheije et al. 2019)	Variable; 17/17; 3 NA	−	Anteverted nares, asymmetric ears with abnormal helix	−	−
Intellectual disability	19/21; 8 NA	20/20	+	+	NA	−

*NA* not available, + phenotype observed, − phenotype not observed, *ASD* atrial septal defect, *SNV* single nucleotide variant, *dels/dups* microdeletions/microduplications, *indels* small insertion/deletions, *pat* paternal, *mo* months, *y* years

^#^Reference [2]—Verheije et al. (2019)

^&^Reference [2]—Verheije et al. (2019) [14],—Douglas et al. (2018), [15]—Giliberti et al. (2020), [18]—Hildebrand et al. (2020), [19]—Su et al. (2020), [20]—Fujita et al. (2016), [21]—Santoro et al. (2021), [22]—Srivastava et al. (2018)

^$^Genomic coordinates are based on the genome assemble GRCh37/hg19

*Exon definition is based on the NM_170676.5 transcript

*MEIS2* mosaicism is rare and has not been frequently documented in the literature. A mosaic 123 kb deletion encompassing only the exon 9 of *MEIS2* was reported to be responsible for a cleft soft palate, ventricular septal defect, and bilateral moderate hearing loss [3]. Su et al*.*, recently reported a boy with the phenotypic spectrum, including atrial and ventricular septal defects, developmental delay, facial dysmorphism, primary neutropenia, branchial anomalies, and complex genital anomalies, inherits a *MEIS2* p.R333del mutation from his unaffected father with a low-level mosaicism [19]. Our study represents the first report showing that multiple family members inherit a genomic deletion resulting in a 3′ *MEIS2* partial truncation from a mosaic father, who is healthy other than an atrial septal defect (ASD). However, the ASD in the father is likely not related to the mosaic 3′ *MEIS2* deletion as mosaicism level can vary significantly among tissues and it was determined only in peripheral blood in this study. In addition, the molecular origin of the mosaicism, as a de novo event arising post-zygotically or a gene conversion following a germline mutation, remains to be defined. These findings would have implications for recurrence risk counseling for families.

Interestingly, a 3′ *MEIS2* deletion only affecting last three exons ([hg19] chr15:37163900_37195301, NSV569198 in the Database of Genomic Variants (DGV)) has been reported in a normal individual, that served as a control used for a study to establish a copy number variation morbidity map of developmental delay [27]. This suggests that phenotype in such individual could be very subtle, if any. This finding points to the importance of offering *MEIS2* gene tests covering both SNVs/indels and microdeletions to individuals with milder bifid uvula and developmental delay. This report also raises the possibility that cryptic or tissue-specific mosaicism of genetic lesions involving the *MEIS2* gene could cause diseases and may pose a diagnostic challenge.

Taken together, it is possible that some 3′ *MEIS2* deletions could produce a truncated partial functional protein and therefore act as a hypomorphic mutation, instead of nullimorphic (complete loss of function), causing a mild phenotype. Further studies that provide experimental evidence, such as RNA or protein analysis, are necessary to confirm partial expression of 5′ *MEIS2* and this hypothesis*. *In vivo functional studies are warranted to define three types of *MEIS2* mutations—dominant negative alleles (missense variants) [14], loss-of-function alleles (whole gene deletion or exonic deletion/duplication involving exon 9 and upstream) [2], and hypothesized hypomorphic alleles (deletions involving exon 10 and downstream) [2, 22, 27]. More clinical cases are needed to establish genotype–phenotype correlation.

## Data Availability

The raw data of CGH and FISH were available upon request.

## Abbreviations

MEIS2: Meis homeobox 2
TALE: Three amino acid loop extension
OFC: Orofacial clefts
CMA: Chromosome microarray
aCGH: Array comparative genomic hybridization
CNVs: Copy number variants
SNP: Single nucleotide polymorphism
FISH: Fluorescence in situ hybridization
PHA: Phytohemagglutinin
BAC: Bacterial artificial chromosome
URMC: University of Rochester Medical Center
IUGR: Intrauterine growth restriction
ECG: Electrocardiogram
SNVs: Single nucleotide variants
Indels: Insertions and deletions
HD: Homeodomain
OMIM: Online Mendelian Inheritance in Man
ID: Intellectual disability
ASD: Atrial septal defect
DGV: Database of Genomic Variants
